# Defective autophagy in vascular smooth muscle cells enhances the healing of abdominal aortic aneurysm

**DOI:** 10.14814/phy2.15000

**Published:** 2021-09-07

**Authors:** Akihiro Mochida, Tomoya Mita, Kosuke Azuma, Yusuke Osonoi, Atsushi Masuyama, Kenichi Nakajima, Hiromasa Goto, Yuya Nishida, Takeshi Miyatsuka, Masako Mitsumata, Hirotaka Watada

**Affiliations:** ^1^ Department of Metabolism & Endocrinology Juntendo University Graduate School of Medicine Tokyo Japan; ^2^ Center for Therapeutic Innovations in Diabetes Juntendo University Graduate School of Medicine Tokyo Japan; ^3^ Center for Identification of Diabetic Therapeutic Targets Juntendo University Graduate School of Medicine Tokyo Japan; ^4^ Division of Pathology Department of Clinical Laboratory Yamanashi Kosei Hospital Yamanashi city Yamanashi Japan

**Keywords:** aneurysm, autophagy, healing, smooth muscle cells

## Abstract

Autophagy is an evolutionarily conserved cellular catabolic process essential for cell homeostasis, and thus its failure is associated with several diseases. While autophagy has been reported to play a role in vascular smooth muscle cells (SMCs) in vascular disorders, its precise role in the pathogenesis of abdominal aortic aneurysm (AAA) has not yet been elucidated. In this study, we investigated the role of SMC autophagy in AAA formation. As a mouse model of AAA, we used control apolipoprotein E‐deficient (*apoeKO*) mice and *Atg7cKO* (SMC‐specific *Atg7*‐deficient mice):*apoeKO* mice administered angiotensin II for 4 weeks. Intriguingly, Kaplan‐Meier curves showed that the survival rates of *Atg7cKO*:*apoeKO* mice were significantly higher than those of *apoeKO* mice. The hematoma area in AAA of *Atg7cKO*:*apoeKO* mice was smaller than in *apoeKO* mice despite the lack of a significant difference in AAA incidence between the two groups. Furthermore, the amount of granulomatous tissues was significantly larger and the collagen‐positive area within AAA was significantly larger in *Atg7cKO*:*apoeKO* mice than in *apoeKO* mice. In accordance with these findings, SMCs cultured from *Atg7cKO* mice showed increased expression of collagens, independent of angiotensin II action. Taken together, our data suggest that defective autophagy in SMCs elicits AAA healing that may underlie the better survival rate under dyslipidemia and angiotensin II infusion.

## Graphical abstract

Defective autophagy in smooth muscle cells enhances the healing of hematoma.

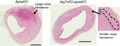

## 1 INTRODUCTION

Abdominal aortic aneurysm (AAA), defined as a focal dilation of the aorta, is a common chronic vascular degenerative disease (Golledge et al., 2006). Excessive dilation of the AAA confers a risk of rupture that frequently causes sudden death. Thus, it is important to prevent AAA and to gain insight into the pathogenic mechanisms of its development and progression in order to identify novel therapeutic targets.

Vascular smooth muscle cells (SMCs) are the major cell component of the arterial wall, and they play important roles in the pathogenesis of AAA (Shen et al., 2020). During AAA development and progression, clonal expansion of a subset of SMCs occurs in the media, and these cells can infiltrate the adventitia. These SMCs show phenotypic switching from contractile cells to phagocyte‐like cells (Shen et al., 2020), which is expected to confer a greater capacity for proliferation, secretion, and migration. This process seems to play an important role in maintaining the integrity of vessel‐ and tissue‐repair processes in response to vascular injury (Owens et al., 2004). On the other hand, these SMCs show increased production of elastolytic enzymes that degrade extracellular matrix components such as elastin and collagen, leading to tissue destruction and a weakened arterial wall (Knox et al., 1997).

Macroautophagy (hereafter referred to as autophagy) is a highly evolutionarily conserved basal cellular process to recycle defective proteins and organelles in order to maintain cell homeostasis. Therefore, defective autophagy has been implicated in various human conditions, including atherosclerotic diseases (Grootaert et al., 2015; Masuyama et al., 2018; Osonoi et al., 2018). In addition, a recent study demonstrated that autophagy‐related genes, including *ATG7*, were increased in the SMCs of human aneurysmal tissue, implicating SMC autophagy in the pathogenesis of AAA (Zheng et al., 2012). In fact, we previously found that medial vulnerability and the fragmentation of elastic fibers were the most important features of the aortic wall during the process of aneurysm formation in mice with SMC‐specific deletion of *ATG7* that were crossed with apolipoprotein E‐deficient (*apoeKO)* mice (Osonoi et al., 2018). Also, a recent study demonstrated that by increasing SMC death and enhancing SMC inflammation, SMC‐specific *ATG5* deficiency promoted the progression of AAA formation in angiotensin II‐treated mice with transforming growth factor‐β (TGF‐β) inhibition (Clement et al., 2019). In contrast, another study reported that SMC‐specific *ATG7* deficiency did not result in dissecting AAA in angiotensin II‐treated mice (Ramadan et al., 2018). Therefore, the pathophysiological role of SMC autophagy in the development and progression of AAA, especially under the condition of angiotensin II infusion, has not yet been fully elucidated.

In this study, we aimed to further investigate the role of SMC autophagy in the development and progression of AAA using *apoeKO* mice infused with angiotensin II as a model of AAA.

## 2 MATERIALS AND METHODS

### 2.1 Animals

*apoeKO* mice and transgelin (*Tagln*) *Cre* ^+/^ *^0^* mice purchased from the Jackson Laboratory (ME, USA) were housed in specific pathogen‐free barrier facilities. *Atg7^f^* ^/^ *^f^* mice were bred with *Tagln Cre* ^+/^ *^0^* mice to generate smooth muscle‐specific *Atg7*‐deficient mice homologous for the floxed allele and hemizygous for the Cre transgene (*Atg7cKO*) (Masuyama et al., 2018; Osonoi et al., 2018). Littermate controls were homogeneous for the floxed allele of the *atg7* gene but did not carry the Cre transgene (*Atg7^f^* ^/^ *^f^*). Then, *Atg7cKO* mice and *Atg7^f^* ^/^ *^f^* were bred with *apoeKO* mice (C57BL/6 background) at a laboratory facility (Charles River Japan, Ibaraki, Japan) to generate the *Atg7cKO*:*apoeKO* mice and *Atg7^f^* ^/^ *^f^*:*apoeKO* mice (control *apoeKO* mice) that were used in this study. Mice were maintained under a 12‐h light/dark cycle and fed a standard rodent diet (CRF‐1, Charles River Japan) with water *ad libitum*. The study protocol was reviewed and approved by the Animal Care and Use Committee of Juntendo University.

### 2.2 Angiotensin II infusion

At the age of 10 weeks, an osmotic minipump (ALZEST, model 1004; DURECT, Cupertino, CA) was implanted under the skin of the back of each mouse after local anesthesia. The skin incision was closed with a wound clip. Angiotensin II at 1,000 ng/kg/min (Sigma‐Aldrich, A9525 Tokyo, Japan) was infused through the osmotic pump that delivered the solution continuously for up to 4 weeks according to the established methodology (Daugherty et al., 2000).

### 2.3 Blood pressure measurements

Blood pressure was measured by a non‐invasive tail‐cuff method using a Model UR‐5000 system (Ueda Co.). The systolic blood pressure was measured at baseline and at 4 weeks after angiotensin II infusion.

### 2.4 Laboratory data

Blood samples were collected when the mice were sacrificed. Lipid concentrations were measured by Skylight Biotech, Inc..

### 2.5 Quantification of atherosclerotic lesions

To evaluate AAA, mice were sacrificed 4 weeks after angiotensin II infusion under anesthesia induced by intraperitoneal injection of sodium pentobarbital. The aorta was flushed with normal saline followed by 10% buffered formalin. The aorta was excised from the root to the common iliac artery, and the connective and adipose tissues were removed. For quantitative analysis of atherosclerotic lesions, the aneurysm‐prone suprarenal portion of the abdominal aorta was embedded in optimal cutting temperature compound, then 4‐μm‐thick cross sections at 50‐μm intervals were prepared with a cryostat. Twelve consecutive sections were taken sequentially, allowed to dry at room temperature for 30 min, and stained with hematoxylin & eosin (Muto Pure Chemicals Co., Ltd., 32042, 30022), Elastica van Gieson (Muto Pure Chemicals, 40322, 40341, 40351, 40362, 40372), or Azan dye (Muto Pure Chemicals). Then, immunohistochemistry was performed using a rat anti‐mouse or human LGALS3/MAC‐2 monoclonal antibody (Cedarlane Laboratories, Ltd., CL8942AP), a mouse anti‐human ACTA2/α‐SMA (smooth muscle actin) antibody (Dako, M0851), type I collagen antibody (abcam Co., Ltd., ab 138492), and type III collagen antibody (abcam Co., Ltd., ab 184993). A commonly used clinical standard to diagnose AAA is an increase in aortic diameter of about 50% (Daugherty et al., 2000). A previous study reported that the average diameter of the normal suprarenal aorta in *apoeKO* mice was 0.8 ± 0.01 mm. Thus, in this study, we defined AAA as a suprarenal aortic diameter larger than 1.25 mm (Wu et al., 2020). The percentage of AAA consisting of hematoma was calculated as the hematoma area divided by the AAA area, multiplied by 100. Berlin blue staining (Muto Pure Chemicals) was used to detect iron, and positively stained areas were counted. The percentages of AAA showing positive staining for α‐SMA, MAC‐2, collagen (via Azan staining), type I collagen, and type III collagen were calculated.

### 2.6 Cell culture

Murine aortic SMCs were isolated as previously described (Osonoi et al., 2018). Mouse aortic SMCs were maintained in Dulbecco's Modified Eagle Medium containing 20% fetal bovine serum, 100 units/mL penicillin, 100 μg/mL streptomycin, 2.5 μg/mL amphotericin B, and 400 μg/mL l‐glutamine. Experiments used cells between passages three and seven, and individual experiments were repeated at least three times with different cell preparations. The medium was changed every 48 h. For all experiments, SMCs were plated on six‐well plates at 10 × 10^4^ cells per well. Cell counts were performed using a hemocytometer on days 2, 4, 6, and 8.

SMCs were cultured with or without angiotensin II at 10 mM for up to 24 h. Cells were then harvested and sonicated on ice and centrifuged at 15,000 × *g* at 4℃ for 15 min. The supernatants were subjected to western blot analysis using the following primary antibodies: guinea pig anti‐human SQSTM1/p62 antibody (Progen Biotechnik GmbH, Germany, GP62‐C), rabbit anti‐MAP1LC3B/LC3 antibody (Sigma‐Aldrich, L7543), type I collagen antibody (abcam Co., Ltd., ab 138492), type III collagen antibody (abcam Co., Ltd., ab 184993), and rabbit anti‐GAPDH (glyceraldehyde‐3‐phosphate dehydrogenase) antibody (Cell Signaling Technology, 2118). Antibodies were visualized as described previously (Osonoi et al., 2018).

### 2.7 Statistical analysis

Differences between two groups were analyzed for statistical significance using the Mann–Whitney *t*‐test for continuous variables or the chi‐square test for categorical variables. Differences between more than three groups were examined by one‐way analysis of variance followed by the Tukey's post hoc test. *P*‐values less than 0.05 were considered to indicate a statistically significant difference between two groups. Results are expressed as the mean ± SEM. Data were analyzed using the Statistical Package for Social Science computer software program, version 18 (SPSS Inc.).

## 3 RESULTS

### 3.1 Angiotensin II‐infused Atg7cKO:apoeKO mice showed reduced premature death

**FIGURE 1:**
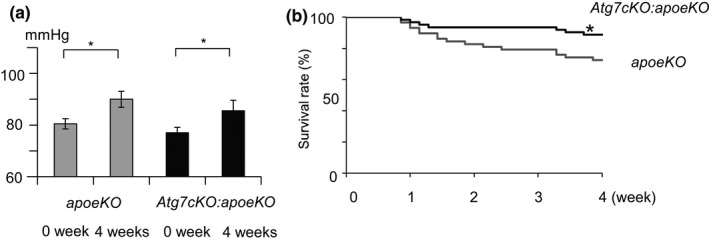
Mean systemic arterial pressure and survival rates of *apoeKO mice* and *Atg7cKO*:*apoeKO* mice infused with angiotensin II. (a) Mean systemic arterial pressure of *apoeKO* mice (n = 21) and *Atg7cKO*:*apoeKO* mice (n = 31) before and after 4‐week angiotensin II infusion. (b) Kaplan‐Meier curves of *apoeKO* mice (n = 62) and *Atg7cKO*:*apoeKO* mice (n = 58) during angiotensin II infusion. *Y*‐axis shows weeks after the beginning of angiotensin II administration. *p < 0.05

To investigate the roles of SMC autophagy in the development and progression of AAA, both *apoeKO* mice (control) and *Atg7cKO*:*apoeKO* mice fed a standard diet at the age of 10 weeks were administered angiotensin II at 1,000 ng/kg/min by osmotic minipump for up to 4 weeks. After 4‐week administration, body weights and lipid levels were comparable between the two groups (Table 1). While both groups showed a significant increase in systolic blood pressure after angiotensin II infusion, there was no significant difference in systolic blood pressure between the two groups at 4 weeks (Figure 1a). Unexpectedly, Kaplan‐Meier curves showed that the survival rates of *Atg7cKO*:*apoeKO* mice were significantly higher than those of *apoeKO* mice (Figure 1b).

**TABLE 1:** Anthropometric data of *apoeKO* and *Atg7cKO*:*apoeKO* mice after 4‐week administration of angiotensin II

	*apoeKO* mice	*Atg7cKO*:*apoeKO* mice	*p* value
Body weight (g)	31.1 ± 0.53 (n = 20)	29.8 ± 0.45 (n = 31)	0.07
Total cholesterol (mmol/L)	13.8 ± 0.6 (n = 20)	12.5 ± 0.4 (n = 37)	0.051
LDL cholesterol (mmol/L)	3.08 ± 0.12 (n = 20)	2.99 ± 0.11 (n = 37)	0.61
HDL cholesterol (mmol/L)	0.85 ± 0.04 (n = 20)	0.81 ± 0.03 (n = 37)	0.41
Triglycerides (mmol/L)	3.92 ± 0.59 (n = 20)	2.88 ± 0.27 (n = 37)	0.12

Blood samples were collected from control *apoeKO* mice and *Atg7cKO*:*apoeKO* mice at 4 weeks after administration of angiotensin II. Each sample was obtained in the fasting state. Data are represented as mean ± SEM.

### 3.2 Angiotensin II‐infused Atg7cKO:Apoeko mice exhibited a smaller hematoma area than apoeko mice

**FIGURE 2:**
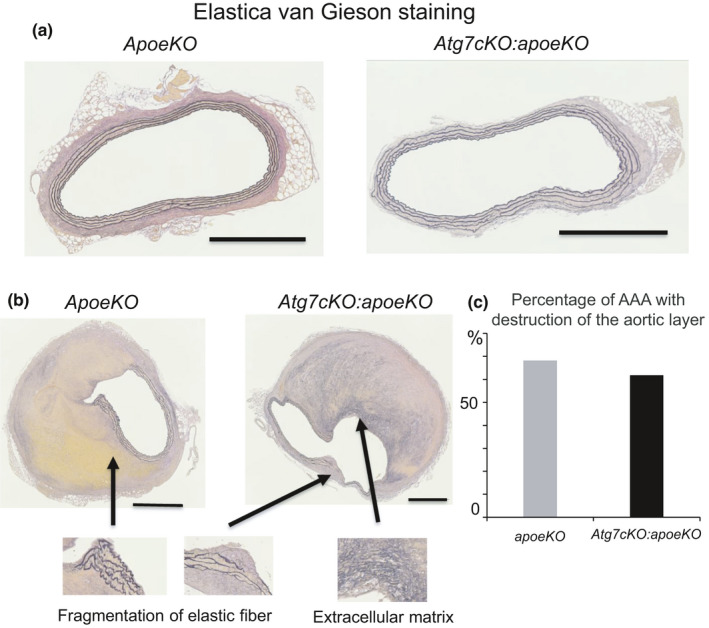
Abdominal aortic aneurysm with normal vascular wall appearance and destruction of the aortic medial layer. (a) Representative histological images of abdominal aorta stained with Elastica van Gieson from control *apoeKO* and *Atg7cKO*:*apoeKO* mice demonstrate a normal vascular wall appearance. Scale bars: 500 μm. (b) Representative histological images of abdominal aorta stained with Elastica van Gieson demonstrate thickening of the aortic wall with the destruction of the aortic medial layer in *apoeKO* and *Atg7cKO*:*apoeKO* mice. (c) Percentage of AAA with the destruction of the aortic medial layer in *apoeKO* (n = 30) and *Atg7cKO*:*apoeKO* mice (n = 43)

**FIGURE 3:**
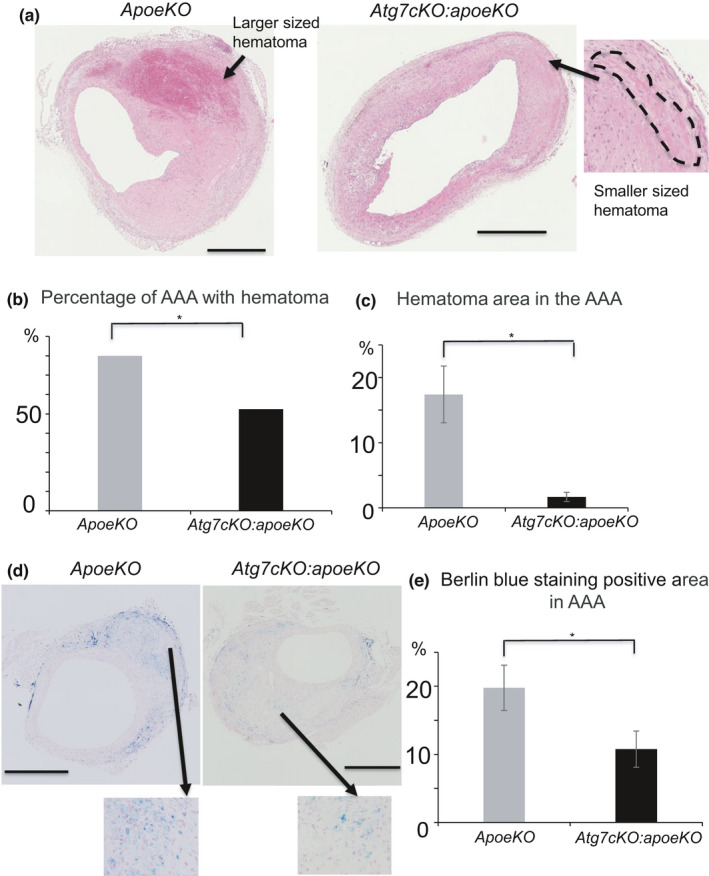
Abdominal aortic aneurysm and hematoma in *apoeKO* and *Atg7cKO*:*apoeKO* mice. (a) Representative histological sections of abdominal aorta stained with hematoxylin and eosin. (b) Percentage of AAA with hematoma and destruction of the aortic medial layer in *apoeKO* (n = 15) and *Atg7cKO*:*apoeKO* mice (n = 21). (c) Area of hematoma in the AAA of *apoeKO* (n = 15) and *Atg7cKO*:*apoeKO* mice (n = 21). Data are shown as the mean ± SEM. *p < 0.05. Scale bars: 500 μm. (d) Representative histological sections of abdominal aorta stained with Berlin blue. (e) Areas of AAA with Berlin blue‐positive staining in *apoeKO* (n = 13) and *Atg7cKO*:*apoeKO* mice (n = 11)

Next, we analyzed the pathological changes in the suprarenal aorta, which is generally regarded as the main site of aneurysm formation (Saraff et al., 2003), by comparing serial cross sections derived from angiotensin II‐infused *apoeKO* mice and *Atg7cKO*:*apoeKO* mice. Based on the definition of AAA as a suprarenal artery diameter larger than 1.5 mm, 22 of 30 (73.3%) *apoeKO* mice and 34 of 43 (79.1%) *Atg7cKO*:*apoeKO* mice developed AAA. There was no significant difference between the *apoeKO* and *Atg7cKO*:*apoeKO* mice in either the incidence of AAA or the suprarenal artery diameter (1,736 ± 86 μm vs 1,667 ± 61 μm, respectively, *p* = 0.24). Among the mice with AAA, seven of 22 (31.8%) *apoeKO* mice and 13 of 34 (38.2%) *Atg7cKO*:*apoeKO* mice had no obvious visual abnormalities of the vascular wall (the typical appearance is shown in Figure 2a). On the other hand, 15 of 22 (68.1%) *apoeKO* mice and 21 of 34 (61.8%) *Atg7cKO*:*apoeKO* mice had thickening of the aortic wall with the destruction of the aortic medial layer (Figure 2b). In these mice, AAA displayed elastic fiber fragmentation and accumulation of extracellular matrix components (Figure 2b). However, there was no significant difference between the two groups in the incidence of AAA with the destruction of the aortic medial layer (Figure 2c). These AAAs are categorized as pseudoaneurysms based on histological analysis. Medial disruption leads to localized extravasation of blood, referred to in this study as a hematoma, that is contained by surrounding connective tissues. Thus, we analyzed the percentages of *apoeKO* mice and *Atg7cKO*:*apoeKO* mice demonstrating hematoma in AAA lesions with accompanying destruction of the aortic medial layer (Figure 3a). The incidence in *apoeKO* mice (12 of 15, 80.0%) was higher than that in *Atg7cKO*:*apoeKO* mice (11 of 21, 52.3%, *p* < 0.01) (Figure 3b). In addition, a larger‐sized hematoma was observed in *apoeKO* mice more frequently than in *Atg7cKO*:*apoeKO* mice (Figure 3a). The area of AAA consisting of hematoma was significantly larger in *apoeKO* mice than in *Atg7cKO*:*apoeKO* mice (Figure 3c). Berlin blue was used to detect the presence of hemorrhage, and positively stained areas were significantly larger in *apoeKO* mice than in *Atg7cKO*:*apoeKO* mice (Figure 3d,e).

### 3.3 Angiotensin II‐infused Atg7cKO:Apoeko mice showed a larger amount of granulation tissue

**FIGURE 4:**
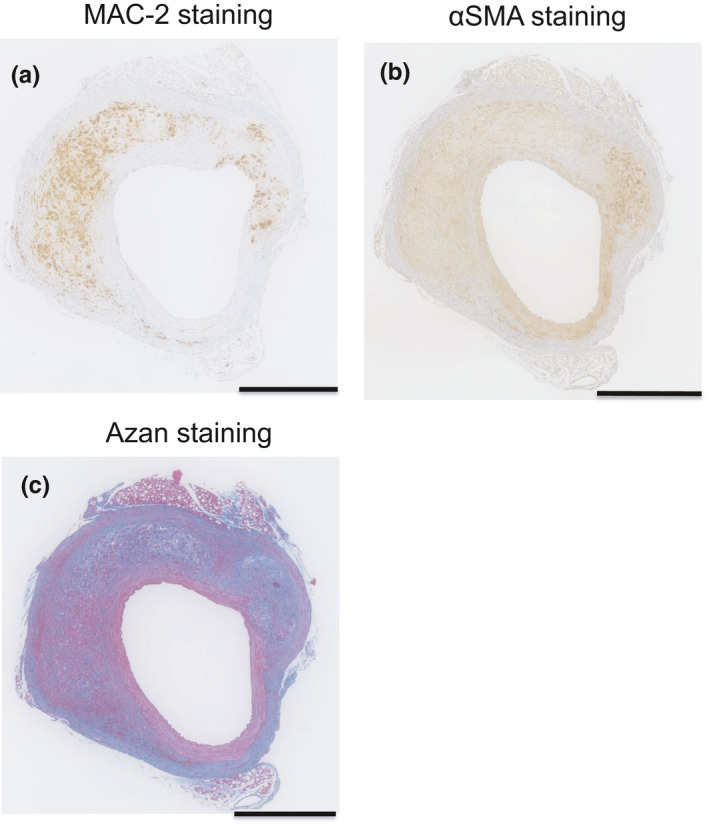
Granulation tissue in the abdominal aorta of an *Atg7cKO*:*apoeKO* mouse consists of macrophages, α‐smooth muscle actin‐positive cells, and collagen. (a) Representative histological sections of the AAA of an *Atg7cKO*:*apoeKO* mouse stained with MAC‐2 antibody. (b) Representative histological sections of the abdominal aorta of an *Atg7cKO*:*apoeKO* mouse stained with ACTA2/α‐SMA antibody. (c) Representative histological sections of the abdominal aorta of an *Atg7cKO*:*apoeKO* mouse stained with Azan dye. Scale bars: 500 μm

**FIGURE 5:**
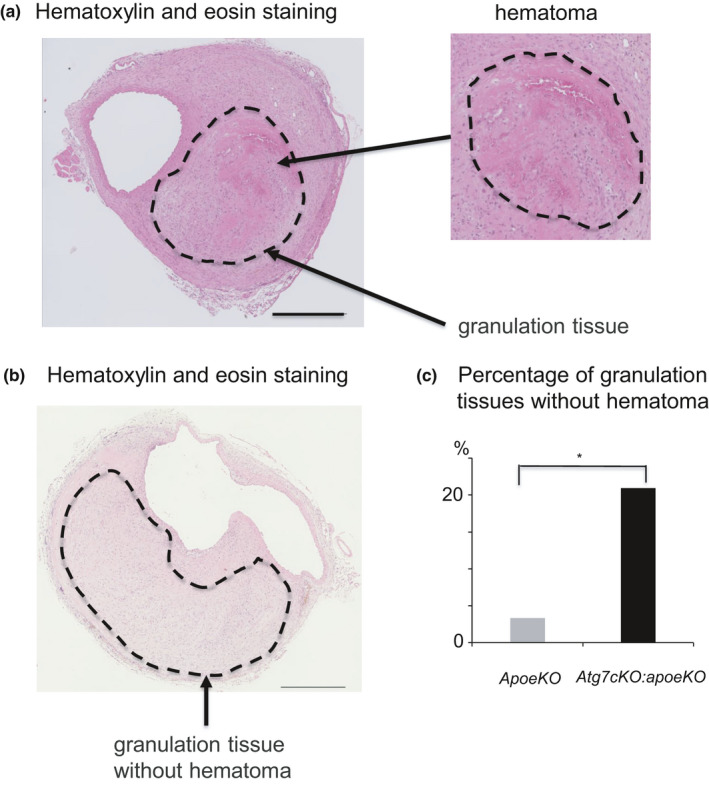
Granulation tissue with a small hematoma in the abdominal aorta of an *Atg7cKO*:*apoeKO* mouse. (a) Representative histological section of the abdominal aorta of an *Atg7cKO*:*apoeKO* mouse stained with hematoxylin and eosin. (b) Representative histological section of the abdominal aorta of an *Atg7cKO*:*apoeKO* mouse stained with hematoxylin and eosin. (c) Percentage of granulation tissue without hematoma in *apoeKO* (n = 30) and *Atg7cKO*:*apoeKO* mice (n = 43). Scale bars: 500 μm

**FIGURE 6:**
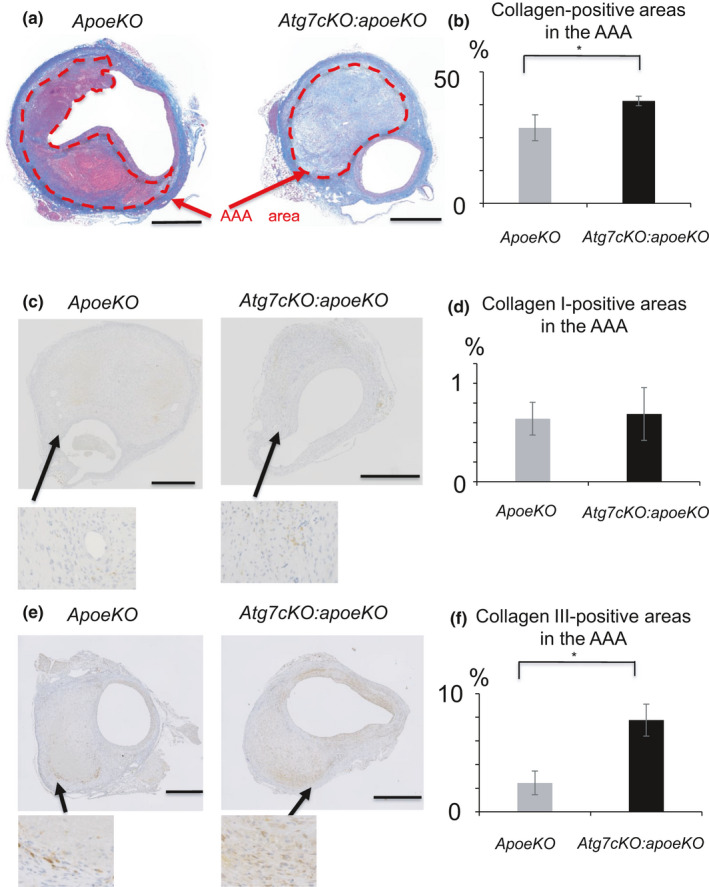
Healing process of hematoma in *apoeKO* and *Atg7cKO*:*apoeKO* mice. (a) Representative histological images of abdominal aorta stained with Azan dye. (b) Percentage of the AAA that is collagen positive in *apoeKO* (n = 13) and *Atg7cKO*:*apoeKO* mice (n = 20). (c) Representative histological images of abdominal aorta stained with type I collagen. (d) Percentage of the AAA that is type III collagen positive in *apoeKO* (n = 13) and *Atg7cKO*:*apoeKO* mice (n = 20). (e) Representative histological images of abdominal aorta stained with type III collagen. (f) Percentage of the AAA that is type I collage positive in *apoeKO* (n = 8) and *Atg7cKO*:*apoeKO* mice (n = 14). Data are shown as the mean ± SEM. *p < 0.05. Scale bars: 500 μm

**FIGURE 7:**
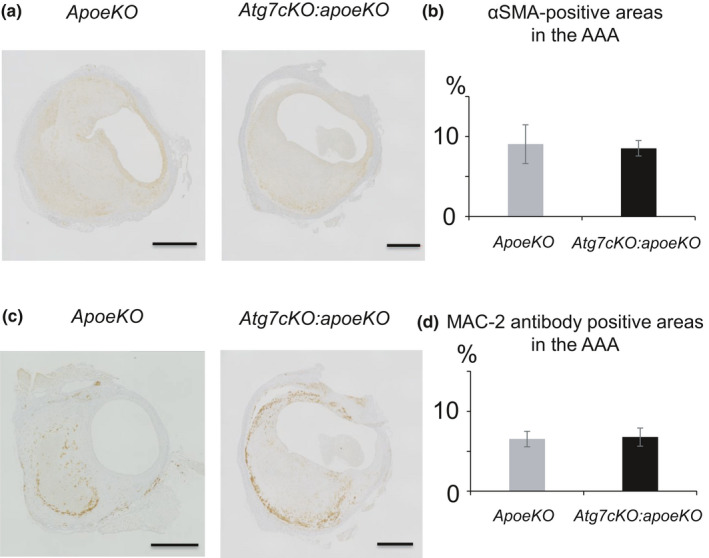
Composition of abdominal aortic aneurysm in *apoeKO* and *Atg7cKO*:*apoeKO* mice. (a) Representative histological images of the AAA of *apoeKO* mice and *Atg7cKO*:*apoeKO* mice. stained with ACTA2/α‐SMA antibody. (b) Areas of α‐SMA‐positive regions in AAA of *apoeKO* (n = 13) and *Atg7cKO*:*apoeKO* mice (n = 20). (c) Representative histological images of AAA of *apoeKO* mice and *Atg7cKO*:*apoeKO* mice stained with MAC‐2 antibody. (d) Areas of MAC‐2 positive regions in AAA of *apoeKO* (n = 13) and *Atg7cKO*:*apoeKO* mice (n = 20). Data are shown as the mean ± SEM. *p < 0.05. Scale bars: 500 μm

Once a local medial disruption has stabilized, the hematoma starts to remodel. At the latest stage of hematoma healing, red blood cells are replaced by SMCs, fibroblasts, and collagen. This process is reflected by the presence of granulation tissue, which contains large numbers of cells, including neutrophilic cells, lymphocytes, macrophages, and myofibroblasts, in addition to capillary vessels. Indeed, immunohistochemistry showed that granulation tissue stained positively for MAC‐2, a macrophage marker (Figure 4a); α‐SMA, a marker of SMCs and myofibroblasts (Figure 4b); and Azan stain, indicating collagen deposition (Figure 4c). In *Atg7cKO*:*apoeKO* mice, granulation tissue with smaller‐sized hematoma was frequently identified (Figure 5a), suggesting enhanced healing of AAA in these mice. Next, we quantitated the percentage of granulation tissue lacking hematoma in both groups and found that it was significantly higher in *Atg7cKO*:*apoeKO* mice (nine of 43, 20.9%) than in *apoeKO* mice (one of 30, 3.3%, *p* < 0.01) (Figure 5b,c). In addition, the area of collagen with positive Azan staining within AAA (Figure 6a,b) was significantly larger in *Atg7cKO*:*apoeKO* mice than in *apoeKO* mice. To investigate what type of collagen may contribute to the healing of AAA, we quantified collagen type I‐ or III‐positive areas within AAA. While there were no significant differences in the percentage of the AAA area that was positive for type I collagen (Figure 6c,d), the percentage positive for type III collagen was significantly larger in *Atg7cKO*:*apoeKO* mice than in *apoeKO* mice (Figure 6e,f). On the other hand, there was no statistically significant difference between the two groups in the percentage of the AAA area that was positive for either α‐SMA (Figure 7a,b) or MAC‐2 (Figure 7c,d). Taken together, these data suggest that defective autophagy in SMCs enhances the healing of hematoma by collagen accumulation within AAA.

### 3.4 Defective autophagy in SMCS increases the expression of collagen independently of angiotensin II action

**FIGURE 8:**
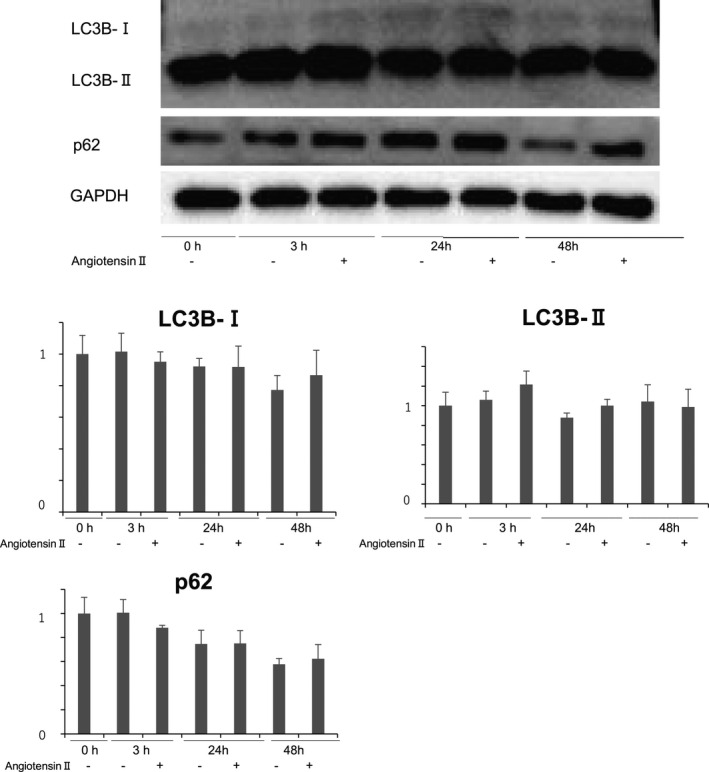
Angiotensin II does not induce autophagy in smooth muscle cells. Smooth muscle cells from control *Atg7^f^* ^/^ *^f^* mice at 10 weeks of age were isolated and exposed to angiotensin II for 3, 24, and 48 h. Western blot analysis was performed using antibodies against LC3B and p62. Representative results from five independent experiments are shown in the upper panel. Quantitative measurements of LC3B‐I, LC3B‐II, and p62 are shown in the lower panel. Data are shown as the mean ± SEM of five independent experiments. The value of the data from smooth muscle cells before angiotensin II administration was set to 1.0

A previous study demonstrated that angiotensin II‐induced autophagy in primary rat aortic vascular SMCs and A7r5 cells (Mondaca‐Ruff et al., 2018). To confirm whether angiotensin II promotes autophagy in mouse SMCs, we isolated primary SMCs from control Atg7^f/f^ mice, then stimulated them with angiotensin II at 100 nM for 3, 24, and 48 h. As shown in Figure 8, we did not find any significant changes in LC‐3B II or p62 expression after adding angiotensin II. Also, there were no statistically significant differences in the expression levels of LC‐3B‐I, LC3B‐II, or p62 between two groups at 3, 24, and 48 h.

**FIGURE 9:**
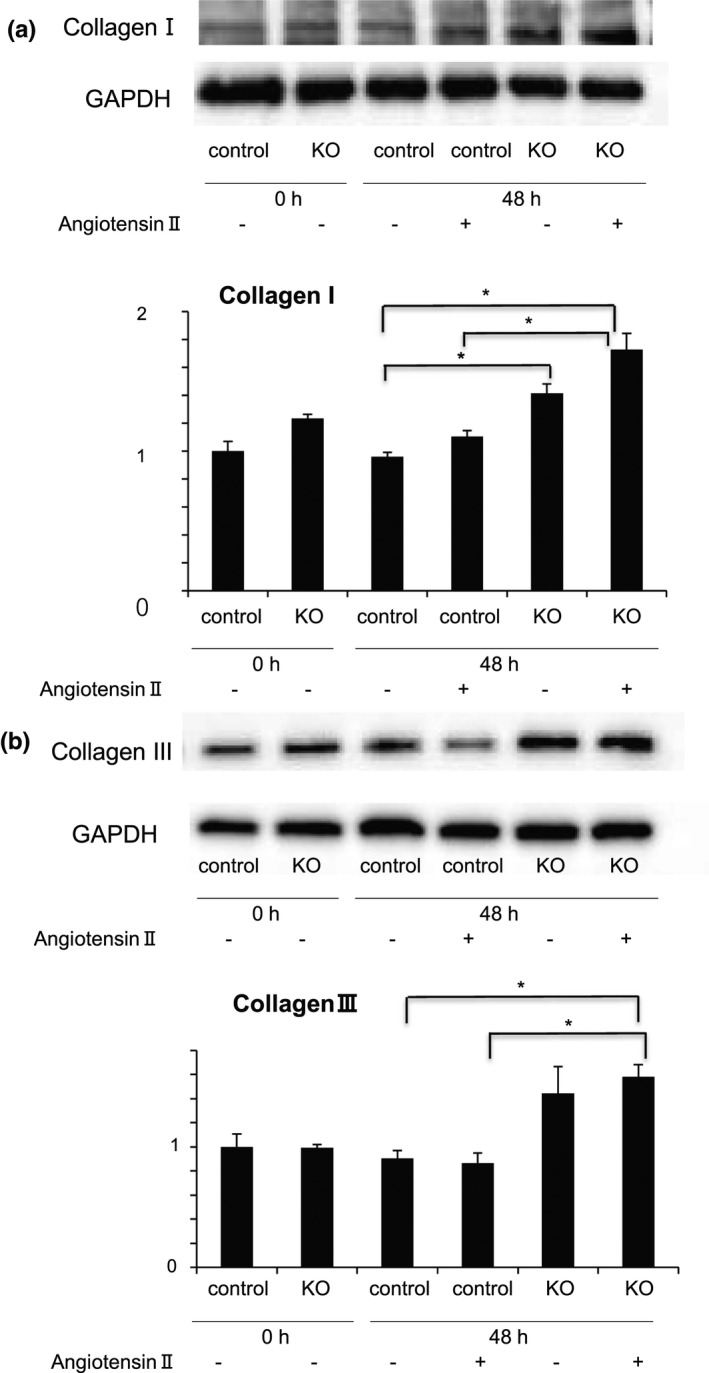
Defective autophagy in smooth muscle cells increases the expression of collagen independent of angiotensin II action. SMCs from control *Atg7^f^* ^/^ *^f^* (control) and *Atg7cKO* mice (KO) at 10 weeks of age were isolated and exposed to angiotensin II for 48 h. Western blot analysis was performed using antibodies against collagen type I (a) and type III (b). Representative results and the quantities of collagen type I (a) and type III (b) are shown. Data are shown as the mean ± SEM of six independent experiments. *p < 0.05. The value of the data from control SMCs at baseline was set to 1.0. SMCs, smooth muscle cells

SMCs are one of the main producers of collagen, and as such, they play an important role in the healing of AAA in the vascular wall. Thus, we investigated the effect of defective autophagy on the expression of type I and III collagen. Angiotensin II administration did not affect the expression levels of either type of collagen, either in SMCs from *Atg7cKO* mice or in those from control *Atg7^f^* ^/^ *^f^* mice (Figure 9a,b). On the other hand, the expression levels of type I collagen in SMCs from *Atg7cKO* mice were significantly higher than those in SMCs from control *Atg7^f^* ^/^ *^f^* mice, regardless of angiotensin II administration (Figure 9a). The same appeared to be true regarding the expression levels of type III collagen (Figure 9b). These data show that defective autophagy in SMCs increases the expression of type I and III collagen independently of angiotensin II action.

## 4 DISCUSSION

This study elucidated the role of SMC autophagy in the pathophysiology of AAA by administering angiotensin II to *apoeKO* mice and *Atg7cKO*:*apoeKO* mice. *Atg7cKO*:*apoeKO* mice infused with angiotensin II had a higher survival rate than *apoeKO* mice, but there was no significant difference in the incidence of AAA between the two groups. On the other hand, hematoma occurred more frequently in *apoeKO* mice than in *Atg7cKO*:*apoeKO* mice. In addition, there was a significantly greater amount of granulation tissue without hematoma in *Atg7cKO*:*apoeKO* mice than in *apoeKO* mice. Furthermore, collagen‐positive areas within AAA were significantly larger in *Atg7cKO*:*apoeKO* mice than in *apoeKO* mice. These data suggest that defective autophagy in SMCs enhances the healing of AAA, and therefore defects in this autophagy may prevent AAA from rupturing.

In this study, we used a mouse model of AAA based on angiotensin II infusion to investigate the role of SMC autophagy in the development and progression of AAA. The AAA induction method was successful, as the AAA incidence and the survival rate of the *apoeKO* mice infused with angiotensin II were almost identical to those in previous studies (Fashandi et al., 2018; Xiao et al., 2020). *Atg7cKO*:*apoeKO* mice that received angiotensin II did not develop AAA more frequently than *apoeKO* mice. Consistent with our finding, another study demonstrated that SMC‐specific deletion of *Atg7* did not cause a higher rate of AAA dissection in angiotensin II‐treated mice than in control *apoeKO* mice (Ramadan et al., 2018). In contrast, a previous study demonstrated that SMC‐specific deletion of *Atg5* promoted AAA formation in angiotensin II‐treated mice with TGF‐β inhibition by increasing SMC death (Clement et al., 2019). Our study did not investigate the reason for this discrepancy, but it may be due to differences in the mouse model of AAA (angiotensin II‐treated *Atg5*‐deficient mice with TGF‐β inhibition vs. angiotensin II‐infused *Atg7*‐deficient mice without TGF‐β inhibition). In fact, angiotensin II‐treated *Atg5*‐deficient mice with TGF‐β inhibition showed a higher incidence and greater severity of AAA than angiotensin II infused *Atg7*‐deficient mice without TGF‐β inhibition. Thus, autophagy in SMCs may play different roles in the pathophysiology of AAA depending on disease stage or severity.

In our previous study, *Atg7cKO*:*apoeKO* mice fed a western diet for 14 weeks showed not only atherosclerosis progression but also an increased frequency of AAA rupture without the administration of any medications, leading to reduced survival (Osonoi et al., 2018). In this study, unexpectedly, *Atg7cKO*:*apoeKO* mice infused with angiotensin II had a higher survival rate than *apoeKO* mice. This discrepancy may be due to differences in the incidence of AAA and aortic rupture between our previous and current studies. In our previous study, local medial destruction due to expanding atherosclerotic lesions under a western diet was likely to be a primary cause of the development of local, saccular, aneurysm‐like dilatations that were commonly observed in the atherosclerotic plaque area of *Atg7cKO*:*apoeKO* mice. Notably, control *apoeKO* mice did not develop such lesions or die of aortic rupture. In our current study, angiotensin II promoted not only the formation of advanced AAA, characterized by local medial destruction and inflammation, but also AAA rupture, even in *apoeKO* mice. At advanced stages of AAA, defective autophagy in SMCs may play an important role in strengthening aortic tissue and limiting the development of AAA rupture by enhancing the production of collagen within AAA.

On the other hand, another study reported that due to aortic rupture, angiotensin II‐treated *Atg5*‐deficient mice with TGF‐β inhibition had a lower survival rate than control mice (Clement et al., 2019). As TGF‐β enhances SMC survival, inhibits matrix degradation, and suppresses vascular inflammatory responses, TGF‐β inhibition causes destruction of the extracellular matrix and structural weakness of the aortic wall in angiotensin II‐infused mice (Wang et al., 2010). In this context, defective autophagy in SMCs results in increased cell death and endoplasmic reticulum stress‐dependent inflammation, leading to increased severity of AAA disease and a higher incidence of fatal AAA dissection (Clement et al., 2019). In addition, a previous study demonstrated that TGF‐β inhibition promoted aneurysmal aortic dilatation and aortic rupture while delaying the healing of AAA (Lareyre et al., 2017). These factors could account for the lower survival rate of angiotensin II‐treated *Atg5*‐deficient mice with TGF‐β inhibition. In contrast, the angiotensin II‐treated *apoeKO* mice used in this study demonstrated a low rate of reproducible severe AAA and AAA rupture. Accordingly, one explanation for the different findings of the previous study (Clement et al., 2019) and our current study is that the role of autophagy may vary depending on disease severity.

As a first step in the healing process (Hoh et al., 2018), circulating neutrophils infiltrate the area containing the hematoma. Then, monocytes and macrophages are recruited to the site of the hematoma to remove extracellular matrix debris by phagocytosis. Finally, proliferative, migrating myoblasts and SMCs promote repair and collagen production, leading to the formation of a collagen‐containing scar. Even in *apoeKO* mice infused with angiotensin II, a previous study demonstrated that red blood cells within the hematoma were replaced by macrophages, SMCs, fibroblasts, collagen, and capillary ingrowth at the latest stage of the hematoma healing process (Trachet et al., 2017). In our study, such granulation tissues were found more frequently in *Atg7cKO*:*apoeKO* mice infused with angiotensin II than in *apoeKO* mice infused with angiotensin II. These data suggest that defective autophagy in SMCs enhances the healing of AAA.

SMCs play an important role in vascular remodeling by producing extracellular matrix components, including collagen. Indeed, collagen synthesis was shown to be increased in the dissected aortic media (Wang et al., 2012). In this regard, defective autophagy in SMCs may increase collagen synthesis. Both Grootaert et al. and our group demonstrated that SMC‐specific *Atg7* deficiency increased the medial thickness and resulted in the development of advanced plaques with increased collagen content (Grootaert et al., 2015; Osonoi et al., 2018). Notably, defective autophagy in SMCs increased collagen synthesis independently of angiotensin II action in the current study. This increased collagen synthesis was associated with a larger area of collagen within AAA in *Atg7cKO*:*apoeKO* mice, as determined by positive Azan staining. In particular, the type III collagen‐positive area in *Atg7cKO*:*apoeKO* mice was significantly larger than that in *apoeKO* mice. A previous study demonstrated that arterial vulnerability of type III collagen‐haploinsufficient mice resulted in aortic rupture in response to angiotensin II (Faugeroux et al., 2013). Thus, in general, the accumulation of collagen within AAA, in particular type III collagen, may increase aortic tissue strength and thereby potentially prevent AAA from rupturing. This in turn may result in a higher survival rate of *Atg7cKO*:*apoeKO* mice. Taken together, the increased production of collagen by migrating SMCs and/or myofibroblasts may promote the healing of AAA in *Atg7cKO*:*apoeKO* mice. On the other hand, there was no significant difference between the two groups in the percentage of AAA that was positive for type I collagen, which is inconsistent with in vitro findings. The protein expression levels of type I collagen within AAA were much lower than those of type III collagen. Although it is unclear how the expression levels of collagen types I and III differ within AAA induced by angiotensin II, a previous study demonstrated that the protein expression levels of type I collagen in advanced plaques were much lower than those of type III collagen (Grootaert et al., 2015). Thus, it may be difficult to detect differences in type I collagen‐positive areas by immunohistochemistry. Also, the mechanisms by which defective autophagy in SMCs enhances collagen production remain unclear at present. In addition, in contrast with a previous study (Mondaca‐Ruff et al., 2018), we could not confirm that angiotensin II‐induced autophagy in SMCs, although the type of cell used in this study was different from those in the prior study. Further studies are needed to clarify how defective autophagy in SMCs contributes to the healing process.

The present study has several limitations. First, there are some differences between human and mouse AAA, although mouse models provide useful clues regarding AAA pathophysiology. Human AAA is located in the infrarenal region, while mouse AAA caused by angiotensin II infusion is localized in the suprarenal region (Bruemmer et al., 2011). The mechanism underlying this discrepancy is unclear, but it may be related to differences involving aortic structure, such as the ratio of collagen to elastin, and regional flow characteristics (Bruemmer et al., 2011). Furthermore, transmural medial rupture occurs within days of administering angiotensin II to mice, and forms a transmural thrombus, while the initiating events in human are unknown (Bruemmer et al., 2011). In this regard, one of the major challenges is obtaining human AAA tissues at an early disease stage. It should be noted that there are similarities between the mouse model of angiotensin II‐induced AAA and human AAA, such as inflammatory cell infiltration, lumen expansion, extracellular matrix fragmentation, and neovascularization (Saraff et al., 2003). Thus, further studies are needed to determine the relevance of angiotensin II‐induced AAA, and until then our findings should be interpreted with caution from the viewpoint of clinical relevance. Second, we could not determine the causes of death of mice in this study, or the locations of aortic rupture, although autopsy of some of the dead mice showed coagulation of blood in the thoracic or abdominal cavity. Further studies are needed to investigate this issue.

In conclusion, we demonstrated that defective autophagy in SMCs enhanced the healing of AAA. Based on the possible roles of SMC autophagy, inhibiting autophagy may help prevent AAA rupture during the disease stage examined in this study.

## CONFLICTS OF INTEREST

The authors have no conflicts of interest relevant to this article.

## AUTHORS’ CONTRIBUTIONS

All authors contributed to the study design and were involved in all stages of writing the manuscript. AM and TM drafted the manuscript. All authors were involved in analyzing and interpreting the data, reviewing and editing the manuscript, and approving the final manuscript. HW is the principal guarantor of this work, has full access to all the data, and takes responsibility for the integrity of the data and the accuracy of the data analysis.
